# ASFmeter: A Portable A-Mode Ultrasound Device for Abdominal Subcutaneous Fat Thickness Measurement

**DOI:** 10.3390/bioengineering12060567

**Published:** 2025-05-26

**Authors:** Hongyang Zhao, Ran Liu, Guangfei Li, Zhou Zhang, Yanxin Wang, Man Ji, Lin Yang, Dongmei Hao

**Affiliations:** 1 College of Chemistry and Life Science, Beijing University of Technology, Beijing 100124, China; 15210534269@163.com (H.Z.);; 2 Beijing Jishuitan Hospital, Beijing 102208, China; 3 Beijing International Science and Technology Cooperation Base for Intelligent Physiological Measurement and Clinical Transformation, Beijing 100124, China; 4 BJUT-UPV Joint Research Laboratory in Biomedical Engineering, Beijing 100124, China

**Keywords:** abdominal subcutaneous fat (ASF) thickness, a-mode ultrasound, body mass index (BMI), measurement

## Abstract

Background: Obesity is a global health concern linked to an elevated risk of chronic diseases. Abdominal subcutaneous fat (ASF) thickness serves as a key indicator for obesity assessment; however, existing measurement methods often lack simplicity and accessibility. Methods: We developed the ASFmeter, a portable, low-cost A-mode ultrasound device designed for rapid ASF thickness measurement. Forty participants underwent ASF thickness assessment using both the ASFmeter and a conventional B-mode ultrasound system, demonstrating strong agreement (R^2^ = 0.94, SEE = 1.72 mm). Statistical analyses evaluated correlations between ASF thickness and body weight, abdominal circumference, and body mass index (BMI). Results: the ASFmeter exhibited high consistency with B-mode ultrasound measurements, confirming its accuracy. Significant variations in ASF thickness were observed across BMI groups, supporting its utility as a reliable obesity indicator. Conclusions: the ASFmeter offers a user-friendly, portable, and cost-effective solution for ASF measurement, facilitating personal health monitoring and obesity-related risk assessment. This innovation holds promise for widespread application in home-based health management.

## 1. Introduction

In today’s sedentary society, abdominal obesity has become a major contributor to obesity-related health issues, including metabolic syndrome, type 2 diabetes, and cardiovascular diseases [1,2,3]. Accurate and accessible measurement of abdominal subcutaneous fat (ASF) is crucial for early health risk assessment and preventive care.

ASF thickness is a recognized health indicator, correlating with metabolic risk factors and supporting the development of targeted interventions to prevent excessive fat accumulation. Regular monitoring can help individuals adjust their lifestyle and reduce the risk of chronic diseases.

Beyond its established association with metabolic risk and obesity, abdominal subcutaneous fat thickness has also been linked to a range of clinical conditions. For example, elevated ASF has been associated with increased severity of obstructive sleep apnea (OSA) [4], due to its role in altering respiratory mechanics and upper airway pressure. In women with polycystic ovary syndrome (PCOS), greater ASF thickness is correlated with insulin resistance and metabolic abnormalities [5]. These findings underscore the broader clinical relevance of ASF assessment and support the development of accessible tools for its measurement.

Several advanced techniques are available for fat measurement, such as magnetic resonance imaging (MRI), computed tomography (CT), and dual-energy X-ray absorptiometry (DEXA), but these methods are expensive, require specialized facilities, or expose users to radiation [6,7,8]. B-mode ultrasound is effective but demands trained operators and clinical settings [9]. Other indirect methods, including underwater weighing, bioelectrical impedance analysis (BIA), and near-infrared spectroscopy (NIRS), are limited by technical and physiological constraints [10,11,12,13,14]. Emerging techniques such as optoacoustic tomography (OAT) have demonstrated the ability to visualize lipid and collagen distributions in human tissue with biochemical specificity [15]. While these approaches offer advanced molecular imaging capabilities, they generally rely on complex instrumentation, high operating costs, and specialized environments, limiting their practicality for routine use. In contrast, A-mode ultrasound offers a simpler and more accessible solution for fat assessment. It provides reliable thickness measurements without the need for high-resolution anatomical imaging, making it particularly well suited for portable and low-cost applications. Despite these advances, a practical, low-cost, and user-friendly device for personal fat assessment remains lacking [16,17].

To address these limitations, this study introduces an innovative device, the ASFmeter, which utilizes A-mode ultrasound technology to measure ASF thickness efficiently and conveniently. Unlike conventional B-mode ultrasound, A-mode ultrasound offers a simpler and more portable approach, making it suitable for both clinical and personal use. The ASFmeter is designed to be user-friendly, cost-effective, and capable of providing real-time fat thickness measurements without the need for specialized training or complex procedures.

To validate its performance, the ASFmeter is systematically compared with a standard B-mode ultrasound device to assess its measurement accuracy and reliability. Additionally, the device is applied in a study to explore the correlation between ASF thickness and various anthropometric parameters, such as body mass index (BMI), waist circumference, and waist-to-hip ratio. By establishing these relationships, the ASFmeter can serve as a valuable tool for monitoring body fat distribution, facilitating early detection of obesity-related risks, and supporting personalized health management.

This novel technology has the potential to transform fat mass assessment by making it more accessible for individuals seeking to proactively understand their body composition. The ASFmeter enables users to better visualize and track their abdominal fat levels, which may increase awareness and support lifestyle adjustments aimed at reducing health risks. Furthermore, the ability to obtain standardized ASF measurements holds great potential for future research exploring the relationship between subcutaneous fat thickness and specific disease outcomes.

## 2. Research and Development of the ASFmeter

### 2.1. Hardware and Framework

**Figure 1:**
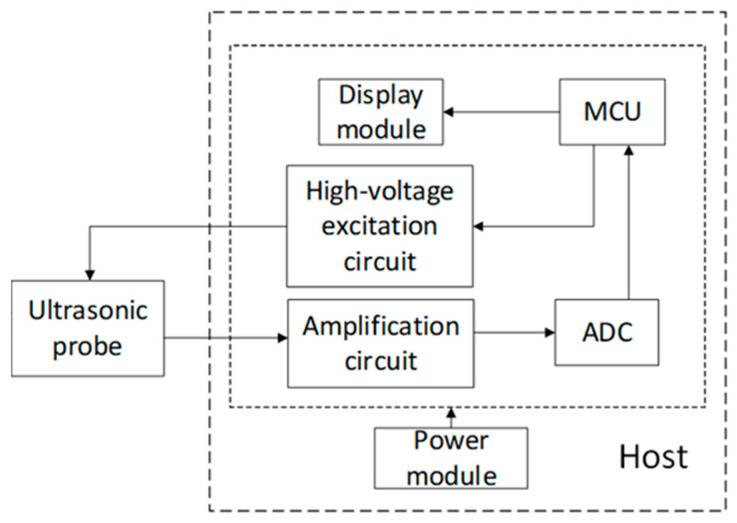
ASFmeter functional block diagram.

As shown in Figure 1, the ASFmeter consists of an ultrasound probe, microcontroller unit (MCU), high-voltage excitation circuit, amplifier circuit, analog-to-digital converter (ADC), display module, and power module.

The ASFmeter utilizes a 2.5 MHz single crystal longitudinal wave ultrasonic transducer for both transmission and reception of acoustic signals. The device operates on a 9 V dry battery power supply. Upon initialization, the MCU module generates complementary 2.5 MHz excitation pulses with the amplitude of 3.3 V. A boost converter elevates the voltage to 7.5 V to drive the high-voltage circuit to produce bipolar excitation pulses for probe activation. The probe converts electrical energy to mechanical waves via the reverse piezoelectric effect. The generated ultrasound propagates through tissue layers including skin, subcutaneous fat, and muscle. Backscattered echoes occur at tissue interfaces with acoustic impedance mismatches. The probe receives echoes and converts them to electrical signals via positive piezoelectric effect. The echo signals are amplified by an amplifier circuit. A 12-bit ADC performs high-speed analog-to-digital conversion at 33.3 MHz. The MCU processes the digital signals to compute ASF thickness. Results are displayed on the integrated display module.

### 2.2. ASF Thickness Calculation

**Figure 2:**
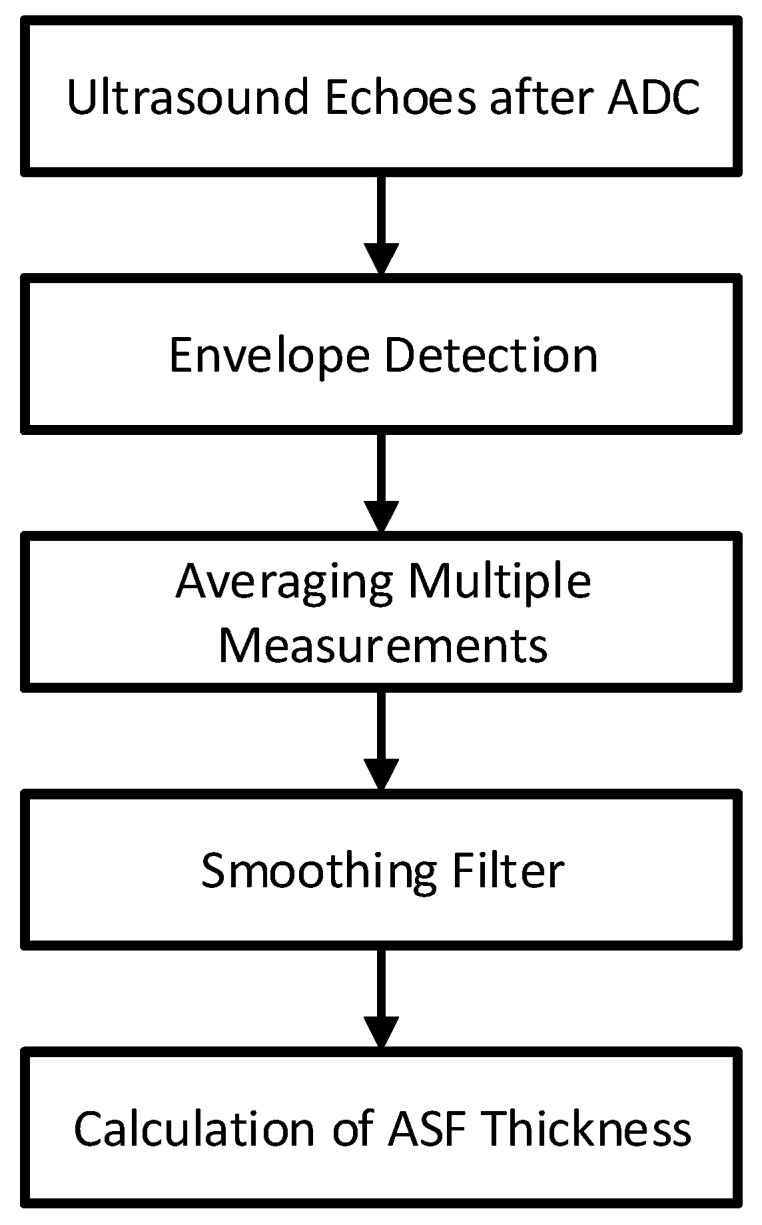
Process of ASF thickness calculation.

Figure 2 shows the processing flow of the ultrasound echo signal.

Following signal acquisition, the ultrasonic echo data underwent advanced processing to determine ASF thickness.

After acquiring the ultrasound echo signals, a 2048-point analysis window was applied to each signal segment. The Hilbert transform was then applied to extract the envelope of the signals. To reduce random noise and improve measurement stability, the envelope extraction process was repeated three times, and the resulting envelopes were averaged. A moving average filter was then employed to smooth the envelope, effectively suppressing high-frequency noise while preserving the prominent echo peaks. Since the deep fascia interface exhibits a significant acoustic impedance mismatch, its corresponding echo typically presents the highest amplitude. By identifying the time point of the maximum peak in the envelope and applying Equation (1), the ASF thickness was calculated.

(1) $$\mathrm{ASF}thickness=\frac{1}{2}\cdot △t\cdot v$$

where △t is the time corresponding to the maximum echo. v is the speed of ultrasound in human soft tissue, with 1540 m/s in this study [18].

**Figure 3:**
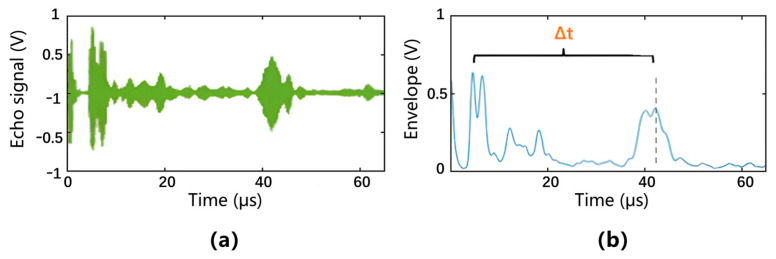
Processing of the echo signal. (**a**) The original echo signal. (**b**) The average envelope of the echo signal.

Figure 3 shows the original echo signal and its corresponding processed envelope, demonstrating clear identification of tissue interfaces. The starting point of the fat layer is determined based on the first significant rebound peak in the A-mode signal, rather than assuming a fixed distance from the skin surface. The end point of the fat layer is determined by the second protruding peak, which usually represents the fat–muscle boundary.

### 2.3. Technical Specifications of the ASFmeter

Table 1 shows the technical specifications of the ASFmeter.

**Figure 4:**
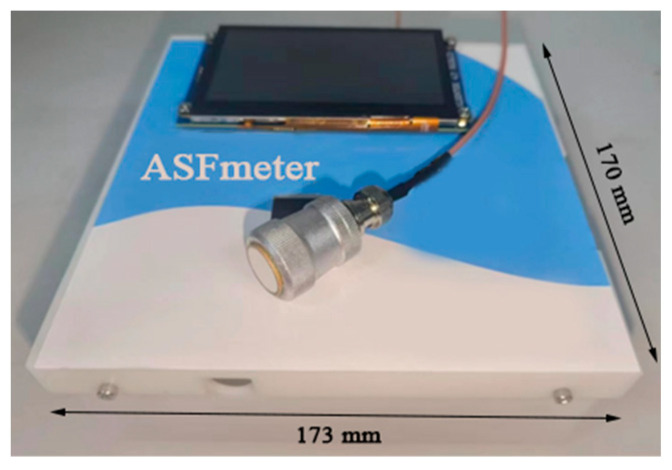
A physical picture of the ASFmeter.

The physical picture of the ASFmeter is shown in Figure 4.

## 3. Measurement with the ASFmeter

### 3.1. Participants

The study recruited 40 healthy college students (21 males, 19 females; mean age 24.3 ± 1.71 years) through standardized screening procedures. Table 2 summarizes the participants’ anthropometric characteristics. Based on World Health Organization BMI classifications, subjects were stratified into three groups:

1. Normal weight (*n* = 28): BMI 18.5–23.9 kg/m^2^;
2. Overweight (*n* = 6): BMI 24.0–27.9 kg/m^2^;
3. Obese (*n* = 6): BMI ≥ 28.0 kg/m^2^.

Prior to participation, all subjects provided written informed consent. The study protocol was conducted in accordance with the ethical principles of the Declaration of Helsinki and received full approval from the Research Ethics Committee of Beijing University of Technology (Ethics Approval No. HS202408001).

### 3.2. Measurement Protocol

Extensive research has established B-mode ultrasound as an accurate and standardized method for subcutaneous fat thickness measurement [19]. In this study, we employed the SSI-3000 color ultrasound Doppler system (SonoScape Co., Ltd., Shenzhen, China) as the reference standard for all measurements.

**Figure 5:**
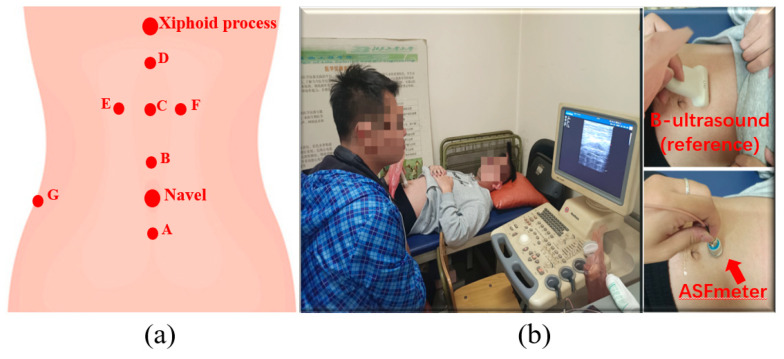
ASF thickness measurement: (**a**) The measurement positions on the abdomen. (**b**) Measurement using the ASFmeter and a B-mode ultrasound device.

Following established protocols [18,20], ASF thickness was measured at seven anatomical locations, as illustrated in Figure 5a:

Position A: 1 cm below the umbilicus;

Position B: 1 cm above the umbilicus;

Position C: Midpoint between umbilicus and xiphoid process;

Position D: 1 cm below xiphoid process;

Position E: 3 cm left of Position C;

Position F: 3 cm right of Position C;

Position G: Intersection of umbilical horizontal line and lateral midline.

Participants assumed a supine position with all measurement positions clearly marked. At each location (A–G), ASF thickness was measured sequentially using the ASFmeter device and the reference B-mode ultrasound system (SSI-3000) with linear array probe, as illustrated in Figure 5b. For both methods, the probe was maintained perpendicular to the skin surface. Five consecutive measurements were obtained and the mean value was recorded as the final ASF thickness. All measurements were performed by a single operator to ensure consistency.

For B-mode ultrasound, the fat thickness was measured manually using the system’s built-in caliper tool by identifying the dermis–fat and fat–muscle interfaces. Each image was measured twice independently, and the results showed minimal differences, indicating good repeatability of the manual measurement process.

### 3.3. Data Analysis

All statistical analyses were performed using IBM SPSS Statistics version 23 (IBM Corporation, New York, NY, USA). Measurement errors and correlation coefficients were calculated to assess agreement between the ASFmeter and the reference B-mode ultrasound device. Bland–Altman analysis was employed to evaluate measurement consistency between the two methods. Pearson/Spearman correlation analysis was conducted to examine relationships between ASF thickness and key anthropometric parameters (height, weight, abdominal circumference, and BMI). Independent samples Mann–Whitney U tests were used to compare ASF thickness at identical anatomical positions across different BMI groups. Intra-group variations in ASF thickness across measurement positions were analyzed using Friedman tests with post-hoc pairwise comparisons.

## 4. Results

### 4.1. Error Analysis

**Figure 6:**
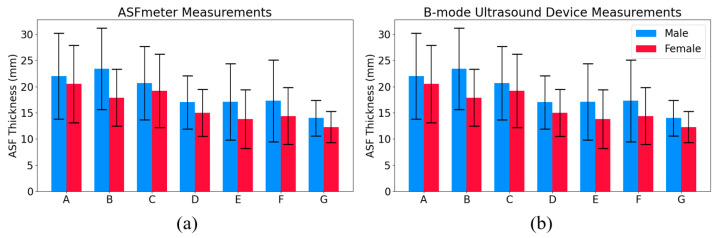
ASF thickness results measured by the ASFmeter and B-mode ultrasound device across seven abdominal positions in male and female participants (positions A–G are illustrated in Figure 5a). (**a**) Results of the ASFmeter. (**b**) Results of the B-mode ultrasound device.

Measurement of ASF thickness at positions A to G in 21 males and 19 females using the ASFmeter and B-mode ultrasound device are shown in Figure 6. Error bars indicate the standard deviation across participants at each position, calculated based on the mean of five repeated measurements per individual. This figure illustrates the distribution of ASF thickness across different anatomical positions for both methods.

Table 3 presents the relative error analysis of ASFmeter measurements, with B-mode ultrasound serving as the reference standard.

### 4.2. Correlation Analysis Between the ASFmeter and B-Mode Ultrasound Device

**Figure 7:**
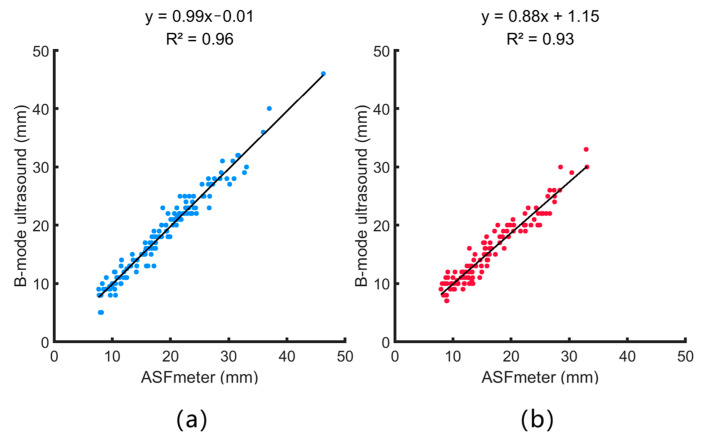
Correlation analysis between ASFmeter and B-mode ultrasound measurements, showing linear regression plots for male and female participants with corresponding R^2^ values. (**a**) Male. (**b**) Female.

Figure 7 shows the correlation between the measurements obtained from the ASFmeter and the B-mode ultrasound device. The linear regression coefficients of determination (R^2^) between these two measures are 0.96 and 0.93 for males and females, and the root mean square (RMS) values of R^2^ are 0.94 mm, indicating a strong correlation between these two measurements. The standard error of the estimate (SEE) for the ASFmeter was determined to be 1.5 mm for males and 1.9 mm for females. The RMS values of SEE are 1.7 mm for both males and females. As a portable device, the ASFmeter demonstrated high performance for measuring ASF thickness.

### 4.3. Bland–Altman Analysis

**Figure 8:**
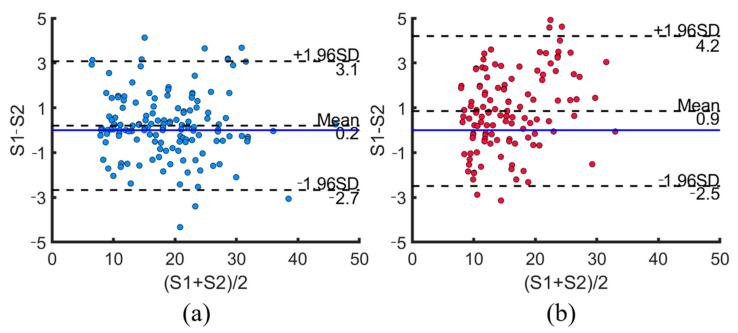
Bland–Altman plots showing agreement between ASFmeter and B-mode ultrasound measurements in male and female participants. (**a**) Male. (**b**) Female.

Figure 8 shows a Bland–Altman plot with the measurements from the ASFmeter (S1) and the B-mode ultrasound device (S2). For the males and females, the mean is about 0.2 and 0.9 above the zero line, and 93.5% and 95.9% of the data are within the upper and lower limits (±1.96SD).

### 4.4. Analysis of ASF Thickness with Anthropometric Parameters

Table 4 presents the correlations between ASF thickness and anthropometric parameters. It can be observed that ASF thickness in both males and females is significantly correlated with weight, AC, and BMI (*p* < 0.01). In addition, ASF thickness is also significantly correlated with height for females (*p* < 0.05) but not for males.

**Figure 9:**
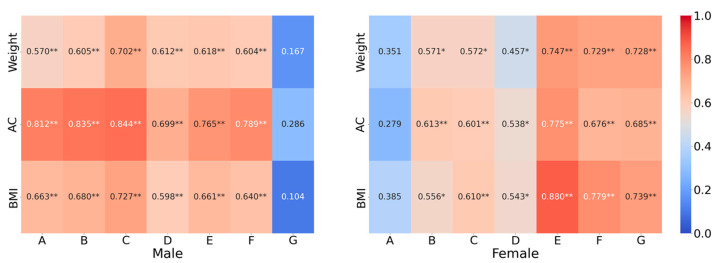
Pearson correlation of ASF thickness with anthropometric parameters. (Note: ** and * indicate significance at the 0.01 and 0.05 levels, respectively (two-tailed)).

Figure 9 presents the correlations between ASF thickness and anthropometric parameters at different positions. It is observed that for males, ASF thickness shows significant correlations with weight, AC, and BMI at positions A to F, but not G. For females, significant correlations were found at positions B to G, but not A.

**Figure 10:**
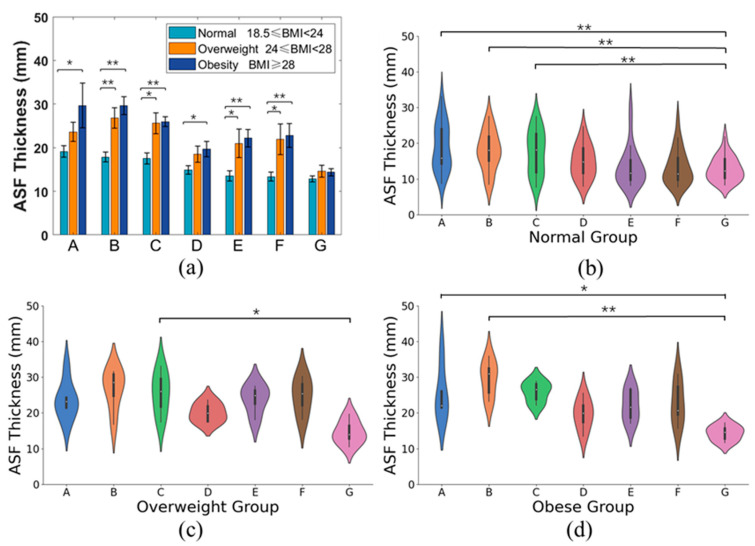
Comparison of ASF thickness between groups and within groups. * *p* < 0.05, ** *p* < 0.01 (**a**) Between groups. (**b**) Within the normal group. (**c**) Within the overweight group. (**d**) Within the obese group.

We compared ASF thickness at the same position between different BMI groups, as shown in Figure 10a. Significant differences were observed between the normal and overweight groups at positions B, C, E, and F, and between the normal and obese groups at all positions except for G. In addition, no significant difference was found between the obesity group and the overweight group at positions A to G, particularly, among these three groups at position G.

We compared ASF thickness at various positions within the same BMI group, as shown in Figure 10b–d. Generally, there was a statistical difference in ASF thickness between positions within the same group. Further, significant differences were found between positions A, B, C, and G in the normal group, between positions C and G in the overweight group, and between positions A, B, and G in the obese group.

## 5. Discussion

The ASFmeter, a portable and user-friendly device, offers a practical solution for personal health monitoring through the self-assessment of ASF accumulation. Regular measurement of ASF thickness can provide individuals with timely feedback on the effects of dietary habits, body weight changes, and physical activity, thereby promoting the adoption of healthier behaviors and lifestyles. Compared with conventional imaging methods such as MRI, CT, and B-mode ultrasound, the ASFmeter is more accessible, affordable, and convenient, making it suitable for both clinical use and home-based applications.

Leveraging A-mode ultrasound technology, the ASFmeter enables accurate and efficient measurement of ASF thickness. Despite being a simpler modality, it demonstrated strong agreement with B-mode ultrasound measurements, showing high correlation, minimal error, and excellent consistency [21]. These results are supported by previous findings indicating that A-mode and B-mode ultrasound can produce comparable measurements of subcutaneous fat thickness in cadaveric specimens [16]. Our study further extends this evidence by confirming similar measurement performance on the human abdomen in vivo.

The seven abdominal positions (A–G) used for ASF thickness measurements in this study were selected based on previous literature, where they are commonly used as standard positions for evaluating abdominal fat distribution. These positions provide anatomical coverage across central and lateral abdominal regions, including areas near the umbilicus, which are known to be highly correlated with metabolic risk. The use of multiple, literature-based positions enhances the representativeness of our measurements and improves their clinical relevance, even though each measurement is localized.

We also observed significant correlations between ASF thickness and key anthropometric parameters, including BMI, abdominal circumference, and weight. These findings are consistent with earlier studies that identified ASF thickness as a reliable health indicator, particularly in relation to metabolic risk factors [3,22]. The ASFmeter’s ability to capture regional differences in ASF thickness across multiple abdominal positions enhances its potential for detailed fat distribution analysis. In our data, participants in the obese and overweight groups exhibited significantly greater ASF thickness in most measurement positions compared to those in the normal group, with the exception of Position G, which is anatomically distant from the umbilical region and showed less variation. Additionally, within each BMI group, ASF thickness varied across positions, with areas near the umbilicus presenting higher values.

Beyond the observed differences in ASF thickness across BMI groups and measurement positions, we also examined measurement variability between male and female participants. We observed gender-based differences in measurement error, with female participants showing higher measurement errors at certain positions, particularly at Position G. Possible explanations include the following: First, the subcutaneous fat layer in the female abdomen is generally thicker [23], making it more difficult to maintain consistent probe pressure and angle on thicker tissue, which may increase measurement variability. Second, the greater compressibility of the female subcutaneous fat layer means that, under the same probe pressure, tissue deformation is likely to be greater than in males, leading to increased variability in echo signal detection. Third, differences in the fibrous structure and tissue heterogeneity within the subcutaneous fat layer may contribute to greater variability in the acoustic signal.

Despite these promising findings, several limitations should be acknowledged. In this study, the ASFmeter was exclusively used to assess abdominal fat, where the subcutaneous layer is relatively thick. Its performance on thinner fat deposits, such as those in the limbs, may be affected by near-field limitations inherent in ultrasound-based techniques [24]. Moreover, physiological factors such as age, sex, and hydration status may influence measurement accuracy. These factors highlight the need for further validation across broader and more diverse populations. In addition, the study population was limited to healthy adults, which may affect the generalizability of the findings to other groups such as elderly individuals, children, individuals with obesity, or patients with chronic diseases. In the future, we plan to conduct research involving a wider range of participants to further validate the applicability of the ASFmeter across different populations.

In terms of practical performance, the specific parameters of the ASFmeter are provided in Table 1, showing that the device can operate continuously for 6 h on a full charge. In addition, the prototype ASFmeter was used continuously over a two-month data collection period without functional failure or noticeable performance degradation, indicating sufficient durability for research and potential clinical applications. However, systematic evaluation of operational parameters such as measurement time, intra-day reproducibility, and long-term durability is still lacking, and we plan to address these aspects in future studies to improve the overall assessment.

In this study, we focused on validating the measurement performance of a novel A-mode ultrasound device, the ASFmeter, for assessing abdominal subcutaneous fat thickness. While ASF thickness is widely recognized as a health-related parameter, our study does not define specific clinical thresholds or intervention targets. Instead, it establishes the technical feasibility and consistency of ASFmeter measurements as a foundation for future research. By enabling standardized and accessible ASF measurement, the ASFmeter contributes to methodological advancement in personal body composition tracking, obesity assessment, and population-level screening.

Future research should focus on expanding the ASFmeter’s applicability and verifying its accuracy against established imaging standards. This includes enrolling participants across a wider range of ages, BMI levels, and health conditions to assess generalizability. Comparative studies with gold-standard imaging modalities such as MRI or CT are also essential to strengthen its clinical validation. While this study does not directly assess disease risk, the existing literature supports the relevance of ASF thickness in conditions such as metabolic syndrome, sleep apnea, and PCOS. The ASFmeter thus provides a reliable means to facilitate future clinical studies in this area. Ultimately, the ASFmeter holds strong potential as a scalable and cost-effective tool for improving obesity management and advancing personal health monitoring.

## 6. Conclusions

In this study, we developed and validated the ASFmeter, a novel A-mode ultrasound device for assessing abdominal subcutaneous fat thickness. The device demonstrated high measurement consistency and strong correlations with standard anthropometric parameters, such as BMI and abdominal circumference, indicating its technical reliability.

While abdominal fat is widely associated with obesity-related health risks, this study does not define specific ASF thickness thresholds for clinical decision-making. Instead, it provides a standardized and accessible tool that lays the groundwork for future research exploring the relationship between subcutaneous fat and metabolic, endocrine, or inflammatory conditions.

The ASFmeter’s simplicity, portability, and cost-effectiveness make it a promising instrument for longitudinal studies, large-scale screenings, and personal health monitoring. Further investigations are needed to establish clinically meaningful cutoff values and to determine how ASF measurements can inform disease risk assessment or intervention planning.

## Figures and Tables

**Table 1:** Parameters of the ASFmeter.

Item	Technical Specifications
Measurement Range (mm)	5~50 mm
Probe Frequency	2.5 MHz
Resolution (mm)	0.6 mm
Input Voltage	6–9 V DC
Power	<5 W
Continuous working hours	6 h
Weight	<1 kg
Dimensions	173 mm × 170 mm × 65 mm

**Table 2:** The anthropometric parameters of the subjects (mean ± std).

Title 1	Male (*n* = 21)	Female (*n* = 19)	Male and Female (*n* = 40)
Height (cm)	178.4 ± 6.5	166.6 ± 7.1	172.8 ± 9.0
Weight (kg)	80.7 ± 10.2	58.0 ± 9.4	70.1 ± 15.0
AC (cm)	88.2 ± 7.8	70.7 ± 7.1	79.9 ± 11.5
BMI (kg/m^2^)	25.4 ± 2.9	20.8 ± 2.3	23.2 ± 3.5

AC: Abdominal circumference.

**Table 3:** Relative measurement error (%) of the ASFmeter compared to B-mode ultrasound at seven anatomical positions (A–G) in male and female participants (mean ± std).

	A	B	C	D	E	F	G
Male (*n* = 21)	4.9 ± 2.3	4.9 ± 2.3	4.2 ± 2.6	6.4 ± 2.4	12.1 ± 4.1	7.5 ± 2.2	8.9 ± 3.7
Female (*n* = 19)	9.7 ± 3.1	11.4 ± 2.8	8.1 ± 2.7	7.9 ± 3.7	13.4 ± 4.5	8.2 ± 3.3	16.2 ± 5.7

**Table 4:** Correlation coefficients between ASF thickness and anthropometric parameters in male, female, and combined groups.

	Male (*n* = 21)	Female (*n* = 19)	Male and Female (*n* = 40)
Height (cm)	0.035	0.219 *	0.217 *
Weight (kg)	0.511 **	0.495 **	0.468 **
AC (cm)	0.664 **	0.497 **	0.528 **
BMI (kg/m^2^)	0.545 **	0.537 **	0.524 **

Note: ** and * indicate significance at the 0.01 and 0.05 levels, respectively (two-tailed).

## Data Availability

The datasets generated and analyzed during the current study are available from the corresponding author on reasonable request. The original data can be provided upon request for peer review purposes.

## Abbreviations

The following abbreviations are used in this manuscript:

BMI	Body mass index
AC	Abdominal circumference
ASF	Abdominal subcutaneous fat
